# Revising cancer incidence in a Central European country: a Hungarian nationwide study between 2011–2019 based on a health insurance fund database

**DOI:** 10.3389/fonc.2024.1393132

**Published:** 2024-10-01

**Authors:** Zoltán Kiss, Tamás G. Szabó, Csaba Polgár, Zsolt Horváth, Péter Nagy, Ibolya Fábián, Valéria Kovács, György Surján, Zsófia Barcza, István Kenessey, András Wéber, István Wittmann, Gergő Attila Molnár, Eszter Gyöngyösi, Angéla Benedek, Eugenia Karamousouli, Zsolt Abonyi-Tóth, Renáta Bertókné Tamás, Diána Viktória Fürtős, Krisztina Bogos, Judit Moldvay, Gabriella Gálffy, Lilla Tamási, Veronika Müller, Zoárd Tibor Krasznai, Gyula Ostoros, Zsolt Pápai-Székely, Anikó Maráz, Gabriella Branyiczkiné Géczy, Lászlóné Hilbert, Láśzló Tamás Berki, György Rokszin, Zoltán Vokó

**Affiliations:** ^1^ MSD Pharma Hungary Ltd, Budapest, Hungary; ^2^ Second Department of Medicine and Nephrology-Diabetes Centre, University of Pécs Medical School, Pécs, Hungary; ^3^ National Institute of Oncology and National Tumor Biology Laboratory, Budapest, Hungary; ^4^ Department of Oncology, Semmelweis University, Budapest, Hungary; ^5^ Department of Oncology, Bács-Kiskun County Teaching Hospital, Kecskemét, Hungary; ^6^ Department of Molecular Immunology and Toxicology and the National Tumor Biology Laboratory, National Institute of Oncology, Budapest, Hungary; ^7^ Department of Anatomy and Histology, HUN-REN–UVMB Laboratory of Redox Biology Research Group, University of Veterinary Medicine, Budapest, Hungary; ^8^ Chemistry Coordinating Institute, University of Debrecen, Debrecen, Hungary; ^9^ RxTarget Ltd., Szolnok, Hungary; ^10^ University of Veterinary Medicine, Budapest, Hungary; ^11^ Department of Deputy Chief Medical Officer II., National Public Health Center, Budapest, Hungary; ^12^ Institute of Digital Health Sciences, Semmelweis University, Budapest, Hungary; ^13^ Syntesia Medical Communications Ltd, Budapest, Hungary; ^14^ Hungarian National Cancer Registry and National Tumor Biology Laboratory, National Institute of Oncology, Budapest, Hungary; ^15^ Department of Pathology, Forensic and Insurance Medicine, Semmelweis University, Budapest, Hungary; ^16^ National Korányi Institute of Pulmonology, Budapest, Hungary; ^17^ 1st Department of Pulmonology, National Korányi Institute of Pulmonology, Budapest, Hungary; ^18^ Department of Pulmonology, Albert Szent-Györgyi Medical Centre, University of Szeged, Szeged, Hungary; ^19^ Department of Pulmonology, Pulmonology Hospital Törökbálint, Törökbálint, Hungary; ^20^ Department of Pulmonology, Semmelweis University, Budapest, Hungary; ^21^ Faculty of Medicine, Department of Obstetrics and Gynecology, University of Debrecen, Debrecen, Hungary; ^22^ Fejér County Szent György, University Teaching Hospital, Székesfehérvár, Hungary; ^23^ Department of Oncotherapy, University of Szeged, Szeged, Hungary; ^24^ Department of Population Statistics, Hungarian Central Statistical Office, Budapest, Hungary; ^25^ Center for Health Technology Assessment, Semmelweis University, Budapest, Hungary; ^26^ Syreon Research Institute, Budapest, Hungary; ^27^ Center for Pharmacology and Drug Research & Development, Semmelweis University, Budapest, Hungary

**Keywords:** cancer burden, cancer incidence, cancer mortality, financial health insurance database, real-wold data, Hungary

## Abstract

**Background:**

The nationwide HUN-CANCER EPI study examined cancer incidence and mortality rates in Hungary from 2011 to 2019.

**Methods:**

Using data from the National Health Insurance Fund (NHIF) and Hungarian Central Statistical Office (HCSO), our retrospective study analyzed newly diagnosed malignancies between Jan 1, 2011, and Dec 31, 2019. Age-standardized incidence and mortality rates were calculated for all and for different tumor types using both the 1976 and 2013 European Standard Populations (ESP).

**Findings:**

The number of newly diagnosed cancer cases decreased from 60,554 to 56,675 between 2011–2019. Age-standardized incidence rates were much lower in 2018, than previously estimated (475.5 vs. 580.5/100,000 person-years [PYs] in males and 383.6 vs. 438.5/100,000 PYs in females; ESP 1976). All-site cancer incidence showed a mean annual decrease of 1.9% (95% CI: 2.4%-1.4%) in men and 1.0% (95% CI:1.42%-0.66%) in women, parallel to mortality trends (-1.6% in males and -0.6% in females; ESP 2013). In 2018, the highest age-standardized incidence rates were found for lung (88.3), colorectal (82.2), and prostate cancer (62.3) in men, and breast (104.6), lung (47.7), and colorectal cancer (45.8) in women. The most significant decreases in incidence rates were observed for stomach (4.7%), laryngeal (4.4%), and gallbladder cancers (3.5%), with parallel decreases in mortality rates (3.9%, 2.7% and 3.2%, respectively).

**Interpretation:**

We found a lower incidence of newly diagnosed cancer cases for Hungary compared to previous estimates, and decreasing trends in cancer incidence and mortality, in line with global findings and the declining prevalence of smoking.

## Introduction

Cancer is the second leading cause of mortality after cardiovascular disease in developed countries (1). Monitoring cancer incidence and mortality is of paramount importance for estimating the burden of the disease as well as for planning and evaluating public health programs and healthcare services (2–4). Although, most of the cancer registries are following the ENCR and IACR rules and work hard to produce incidence figures that are comparable between countries, minor methodological, quality and accuracy differences still exist which limit cross-country comparisons. This is especially an issue in some Central Eastern European countries where cancer registries are underfunded. Hungary has been reported to have the highest cancer incidence rates in Europe (5, 6). Although the National Cancer Registry has been operating according to international guidelines since 2000, its registered data have not been used for such estimates. The overestimation of cancer incidence in Hungary may partly be due to one of the highest reported cancer mortality rates among European countries, since most studies calculate incidence rates based on mortality data (5). However, a recent publication suggested that mortality rates may be significantly influenced by the high autopsy rate of the country resulting in a significant number of post-mortem diagnoses for cancers which may remain asymptomatic for a long time such as lung, colorectal and pancreatic cancers (7). The Hungarian Undiagnosed Lung Cancer (HULC) study showed that 11.1% of reported lung cancer deaths were diagnosed post-mortem by a pathologist in 2019, which is in line with Hungary’s outstandingly high autopsy rate compared to other European countries (8).

The main objective of our nationwide, retrospective study (HUN-CANCER EPI-study – Multiple Cancer Epidemiology study) was to estimate the incidence of all cancer types for the first time in Hungary, using data from the Hungarian National Health Insurance Fund (NHIF) database. Furthermore, we sought to examine the incidence of the top ten most frequent cancer types by sex and age and describe trends in incidence rates for cancers frequently included in European cancer incidence reports, mostly by Ferlay et al. (3) Finally, we aimed to compare our results to findings from other European countries.

## Materials and methods

### Data sources

Our nationwide, retrospective study was performed using the databases of the Hungarian National Health Insurance Fund (NHIF) and the Hungarian Central Statistical Office (HCSO). The NHIF database encompasses almost the entire Hungarian population, including details on drug prescriptions, hospital admissions, outpatient consultations, and medical interventions. It also contains medical information related to diagnosis codes, according to the International Statistical Classification of Diseases and Related Health Problems 10^th^ Revision (ICD-10) (9). The HCSO database is the official source of statistics on annual mortality rates among Hungarian citizens, stratified by the reported cause of death, age, and sex.

Our current study focused on patients diagnosed with any type of cancer (ICD-10 codes C00-97, excluding C44) between January 1, 2011, and December 31, 2019. The identification of records from different sources was based on social security numbers (‘TAJ’ number in Hungarian). To calculate annual cancer incidence rates, the NHIF database was queried for individuals having a cancer-related ICD-10 code in at least two separate reimbursement records. Patients who died within 60 days of the first reported ICD-10 code of interest were also included. If a patient had two or more different cancer-related ICD-10 codes, the ICD-10 code group with a higher number of associated occurrences was considered. For instance, if both the breast cancer-related ICD-10 code C50 and the lung cancer-related C34 code appeared in the reports, but more reimbursement entries were related to C50, the patient was defined as having breast cancer. This approach helped to exclude coding mistakes (e.g., metastasis of breast cancer in the lung coded as primary lung cancer, as the NHIF database is not a medical registry but a reimbursement-focused database). The date of diagnosis was defined as the first appearance of the identified cancer-related ICD-10 code. Second or multiple primary malignancies were excluded from further analysis, the consequences of which are detailed in the limitation section. When defining the ‘dominant’ tumor type in patients with multiple cancer types, only ICD-10 codes with at least two occurrences were considered. To allow for international comparisons, we clustered patients into the following groups in line with Ferlay’s publications (2, 3): lip, oral cavity and pharynx (C00-14), esophagus (C15), stomach (C16), colorectum (C18-21), liver (C22), gallbladder (C23-24), pancreas (C25), larynx (C32), lung (C33-34), melanoma of the skin (C43), breast (C50), cervix (C53), corpus uteri (C54), ovarian (C56), prostate (C61), testicular (C62), kidney (C64-65), bladder (C67), cancer of brain and CNS (C70-72), thyroid (C73), Hodgkin tumor (C81), non-Hodgkin lymphoma (C82-86, C96), multiple myeloma (C88+C90), and leukemia (C91-95).

Patients who did not have any of the previously described ICD-10 codes but had an ICD-10 code starting with C (except for C44) were classified as having other cancer. ICD-10 codes of C44 (non-melanoma skin cancer) were excluded in accordance with international cancer epidemiology studies (2, 3). We also calculated the incidence of any type of cancer for cross-country comparisons as the sum of the number of individuals in all categories (excluding C44).

A screening period was set from 2009 to 2010 to accurately identify newly diagnosed cancer patients from 2011 and to exclude patients with prevalent cancers at the start of the time window who had a prior cancer diagnosis code. To test the sensitivity of our definitions in the query, we carried out multiple calculations based on slightly modified alternative definitions of patients with cancer and compared the results. Incidence rates estimated based on these alternative definitions are included as **Supplementary Material** (**Supplementary Table 1**). The tested definitions included more stringent conditions, specifying a maximum gap between the two closest visits (30-180 days or 30-365 days), as well as less strict requirements, such as requiring only one occurrence of a cancer-related ICD-10 code for the most liberal definition. To identify the most accurate definition for cancer incidence, we investigated all reimbursed cancer-related interventions (systemic anticancer therapy, radiotherapy, morphology code, and cancer-related surgical intervention) for all the previously detailed scenarios (**Supplementary Table 2**). Based on these sensitivity analyses, we prioritized the scenario for cancer definition which required the occurrence of minimum two cancer-related ICD-10 codes of a given cancer type (except for cervical cancer, where a stricter definition was prioritized with a minimum of two cancer-related ICD-10 codes repeated within 30 to 180 days based on the sensitivity analyses in **Supplementary Tables 2**-**4**).

Hungarian population sizes for incidence calculations by age and sex, as well as dates and numbers of cause-specific mortality among cancer patients were obtained from the HCSO (10). For the calculation of incidence rates, annual numbers of newly diagnosed patients with any type of cancer were given as crude numbers (n); new cases were counted for each calendar year (between January 1 and December 31). Annual incidence rates are expressed as standardized rates (per 100,000 person-years [PYs]). Age-standardized incidence per 100,000 PYs were also calculated by sex using the cohort weights from European Standard Population (ESP) 2013 (11) for local trend analyses and ESP 1976 for cross-country comparisons (as most of the prior publications used this standard population) (12). Where crude numbers of any parameter were recorded below ten, we indicated “<10” as the NHIF data protection rule does not allow the presentation of case numbers below ten in a stratum. In these cases, calculations were run on the exact crude numbers.

Aggregated cause-specific mortality data by age and sex was obtained from the HCSO for the disease groups described previously. We considered the number of patients who died of any type of cancer (excluding C44) between January 1 and December 31 of a given year as the number of cancer-related cause-specific deaths. Cancer-specific mortality was expressed as crude numbers (n) and standardized rates per 100,000 PYs. We used standardized incidence and cause-specific mortality rates to evaluate temporal trends in incidence and mortality. Where the crude number of individuals in a category was below three (no more detail can be disclosed to protect the privacy of these patients), the numerical value was imputed to be 1.5 in calculations concerning the data point. The size of the Hungarian population by year, age and sex was also provided by the HCSO.

The study was approved by the National Ethical Committee (IV/298-2/2022/EKU).

### Statistical analysis

Age-standardized incidence and mortality rates per 100,000 PYs by sex were calculated using ESP 2013 (11) cohort weights for local trend analyses and ESP 1976 weights (12) for comparability with Ferlay’s publications (3). Annual trends for incidence and mortality were estimated based on a Poisson-regression model incorporating age and sex as explanatory variables in addition to the year and the log of the population size as offsets. To compensate for the bias potentially introduced by the interdependence of population sizes in consecutive years, 95% confidence intervals (95% CI) were estimated using the robust sandwich method offered by the R package sandwich (13). Differences were considered statistically significant when p-value proved to be lower than 0.05. All calculations were carried out in R v4.2.1 (Available from: https://www.r-project.org).

## Results

### Number of newly diagnosed cases

We identified 60,554 and 56,675 newly diagnosed cancer cases in the NHIF database in 2011 and in 2019, respectively (30,154 in males and 30,400 in females in 2011; 27,970 in males and 28,705 in females in 2019) (**Table 1**). The five most common tumor types in men were as follows (based on the number of newly diagnosed cases in 2019): colorectal cancer (C18-21: n=5,293), lung cancer (C33-34: n=4,787), prostate cancer (C61: n=4,022), bladder cancer (C67: n=1,797), and lip, oral cavity, and pharyngeal cancers (C00-14: n=1,509). In women, the most common tumor types were breast cancer (C50: n=7,305), colorectal cancer (C18-21: n=3,978), lung cancer (C33-34: n=3,711), uterine cancer (C54: n=1,463), and melanoma of the skin (C43: 1,180). The number of newly diagnosed cancers (a) and the number of cause-specific deaths (b) by different age groups are shown in **Supplementary Table 3**. For the sensitivity analyses, newly diagnosed cancer cases based on alternative cancer definitions are shown in**Supplementary Tables 1A–D**.

**Table 1:** Number of newly diagnosed cases and deaths by cancer type, sex, and year in Hungary, HCSO: Hungarian Central Statistical Office; NHIF: National Health Insurance Fund.

A) Incidence of cancer in Hungary	2011			2012			2013			2014			2015			2016			2017			2018			2019		
	Male	Female	Total	Male	Female	Total	Male	Female	Total	Male	Female	Total	Male	Female	Total	Male	Female	Total	Male	Female	Total	Male	Female	Total	Male	Female	Total
**All sites (C00-97 excl. C44)**	**30,154**	**30,400**	**60,554**	**28,971**	**29,409**	**58,380**	**29,584**	**30,133**	**59,717**	**29,529**	**30,015**	**59,544**	**29,069**	**29,961**	**59,030**	**29,251**	**30,241**	**59,492**	**28,969**	**29,358**	**58,327**	**28,014**	**29,075**	**57,089**	**27,970**	**28,705**	**56,675**
Bladder cancer (C67)	1,906	896	2,802	1,969	907	2,876	1,862	898	2,760	1,968	868	2,836	1,922	880	2,802	1,821	890	2,711	1,896	855	2,751	1,814	815	2,629	1,797	866	2,663
Breast cancer (C50)	72	7,775	7,847	81	7,363	7,444	72	7,447	7,519	96	7,565	7,661	71	7,491	7,562	72	7,694	7,766	85	7,511	7,596	80	7,540	7,620	92	7,305	7,397
Cancer of brain and CNS (C70-72)	585	550	1,135	536	530	1,066	563	509	1,072	548	538	1,086	518	480	998	501	446	947	492	483	975	504	475	979	489	424	913
Cancer of lip, oral cavity and pharynx (C00-14)	1,883	615	2,498	1,813	588	2,401	1,787	616	2,403	1,720	618	2,338	1,647	635	2,282	1,749	590	2,339	1,560	582	2,142	1,490	580	2,070	1,509	552	2,061
Cancer of the corpus uteri (C54)		1,370	1,370		1,277	1,277		1,297	1,297		1,368	1,368		1,364	1,364		1,534	1,534		1,367	1,367		1,431	1,431		1,463	1,463
Cervical cancer (C53)		1,067	1,067		978	978		997	997		1,042	1,042		1,043	1,043		1,032	1,032		889	889		863	863		822	822
Colorectum cancer (C18-21)	5,221	4,396	9,617	5,016	4,218	9,234	5,203	4,236	9,439	5,101	4,111	9,212	5,064	4,126	9,190	5,198	4,068	9,266	5,171	3,982	9,153	4,951	3,961	8,912	5,293	3,978	9,271
Gallbladder cancer (C23-24)	205	399	604	226	400	626	246	380	626	263	390	653	232	370	602	231	361	592	235	324	559	250	305	555	199	286	485
Hodgkin tumor (C81)	143	131	274	119	126	245	121	115	236	157	132	289	126	116	242	147	109	256	113	103	216	116	129	245	113	91	204
Kidney cancer (C64-65)	1,095	855	1,950	1,137	811	1,948	1,105	869	1,974	1,103	819	1,922	1,142	834	1,976	1,176	803	1,979	1,125	796	1,921	1,149	783	1,932	1,081	817	1,898
Larynx cancer (C32)	742	142	884	671	121	792	700	126	826	677	125	802	629	117	746	652	111	763	587	95	682	580	89	669	546	89	635
Leukemia (C91-95)	967	952	1,919	952	888	1,840	991	920	1,911	975	964	1,939	990	859	1,849	958	906	1,864	1,034	915	1,949	950	829	1,779	950	817	1,767
Liver cancer (C22)	615	346	961	612	336	948	616	310	926	590	315	905	647	338	985	626	342	968	623	335	958	624	301	925	659	326	985
Lung cancer (C33-34)	6,226	3,681	9,907	5,936	3,698	9,634	5,643	3,809	9,452	5,747	3,703	9,450	5,525	3,821	9,346	5,515	3,788	9,303	5,515	3,875	9,390	5,263	3,748	9,011	4,787	3,711	8,498
Melanoma of the skin (C43)	958	1,018	1,976	896	1,113	2,009	975	1,125	2,100	1,054	1,180	2,234	1,128	1,268	2,396	1,112	1,241	2,353	1,154	1,209	2,363	1,020	1,185	2,205	1,122	1,181	2,303
Multiple myeloma (C88+C90)	239	324	563	242	359	601	269	323	592	293	331	624	262	331	593	264	328	592	296	344	640	317	343	660	268	327	595
Non-Hodgkin lymphoma (C82-86, C96)	717	838	1,555	717	799	1,516	783	967	1,750	778	995	1,773	781	841	1,622	759	923	1,682	775	863	1,638	800	924	1,724	855	1,018	1,873
Oesophagus cancer (C15)	476	127	603	471	133	604	444	124	568	494	124	618	456	116	572	469	108	577	404	99	503	442	90	532	430	100	530
Ovarian cancer (C56)		1,044	1,044		1,052	1,052		1,117	1,117		1,024	1,024		1,062	1,062		1,036	1,036		1,022	1,022		1,005	1,005		936	936
Pancreas cancer (C25)	981	986	1,967	955	992	1,947	998	1,118	2,116	964	1,043	2,007	1,007	1,070	2,077	1,024	1,122	2,146	1,082	1,085	2,167	991	1,082	2,073	1,014	1,080	2,094
Prostate cancer (C61)	3,934		3,934	3,582		3,582	4,239		4,239	4,108		4,108	3,982		3,982	3,996		3,996	3,971		3,971	3,876		3,876	4,022		4,022
Stomach cancer (C16)	1,112	865	1,977	1,074	841	1,915	1,073	828	1,901	980	762	1,742	986	774	1,760	978	746	1,724	965	698	1,663	823	662	1,485	862	598	1,460
Testicular cancer (C62)	659		659	606		606	530		530	580		580	576		576	594		594	539		539	576		576	510		510
Thyroid cancer (C73)	130	465	595	122	454	576	144	546	690	133	536	669	167	528	695	180	597	777	181	519	700	156	538	694	199	607	806
Other	1,288	1,558	2,846	1,238	1,425	2,663	1,220	1,456	2,676	1,200	1,462	2,662	1,211	1,497	2,708	1,229	1,466	2,695	1,166	1,407	2,573	1,242	1,397	2,639	1,173	1,311	2,484
B) Mortality of cancer in Hungary	2011			2012			2013			2014			2015			2016			2017			2018			2019		
	Male	Female	Total	Male	Female	Total	Male	Female	Total	Male	Female	Total	Male	Female	Total	Male	Female	Total	Male	Female	Total	Male	Female	Total	Male	Female	Total
**All sites (C00-97 excl. C44)**	**17,875**	**14,607**	**32,482**	**18,166**	**14,846**	**33,012**	**17,728**	**14,843**	**32,571**	**17,653**	**14,889**	**32,542**	**17,552**	**15,047**	**32,599**	**17,900**	**14,862**	**32,762**	**17,596**	**15,019**	**32,615**	**17,517**	**14,875**	**32,392**	**17,131**	**14,648**	**31,779**
Bladder cancer (C67)	655	268	923	714	269	983	637	262	899	586	320	906	679	280	959	683	292	975	667	316	983	706	302	1,008	713	320	1,033
Breast cancer (C50)	21	2,138	2,159	27	2,096	2,123	27	2,167	2,194	26	2,107	2,133	30	2,220	2,250	25	2,223	2,248	15	2,123	2,138	22	2,127	2,149	26	2,174	2,200
Cancer of brain and CNS (C70-72)	271	320	591	344	296	640	343	322	665	330	332	662	352	336	688	342	326	668	348	314	662	349	339	688	348	276	624
Cancer of lip, oral cavity and pharynx (C00-14)	1,213	281	1,494	1,225	311	1,536	1,146	285	1,431	1,157	303	1,460	1,167	305	1,472	1,122	273	1,395	1,035	298	1,333	1,037	279	1,316	1,018	293	1,311
Cancer of the corpus uteri (C54)		292	292		287	287		275	275		326	326		264	264		317	317		315	315		310	310		331	331
Cervical cancer (C53)		414	414		426	426		405	405		420	420		476	476		396	396		377	377		408	408		386	386
Colorectum cancer (C18-21)	2,835	2,219	5,054	2,810	2,274	5,084	2,865	2,242	5,107	2,848	2,202	5,050	2,825	2,183	5,008	2,845	2,214	5,059	2,816	2,169	4,985	2,836	2,198	5,034	2,837	2,096	4,933
Gallbladder cancer (C23-24)	215	428	643	229	434	663	219	418	637	227	384	611	219	365	584	240	352	592	214	355	569	240	347	587	230	308	538
Hodgkin tumor (C81)	30	13	43	9	22	31	21	18	39	25	10	35	24	22	46	20	15	35	18	21	39	13	24	37	20	15	35
Kidney cancer (C64-65)	459	352	811	487	267	754	456	352	808	486	313	799	466	281	747	475	311	786	469	342	811	470	304	774	458	311	769
Larynx cancer (C32)	490	68	558	484	56	540	476	68	544	445	65	510	389	74	463	409	61	470	398	67	465	395	68	463	471	52	523
Leukaemia (C91-95)	474	474	948	480	435	915	456	453	909	482	404	886	489	470	959	460	439	899	461	404	865	476	479	955	496	397	893
Liver cancer (C22)	516	280	796	538	283	821	553	279	832	625	279	904	565	297	862	581	269	850	590	305	895	565	290	855	578	281	859
Lung cancer (C33-34)	5,558	2,975	8,533	5,763	3,133	8,896	5,418	3,173	8,591	5,456	3,277	8,733	5,356	3,397	8,753	5,542	3,341	8,883	5,393	3,447	8,840	5,341	3,375	8,716	4,997	3,450	8,447
Melanoma of the skin (C43)	214	154	368	210	169	379	186	163	349	196	186	382	216	135	351	201	142	343	190	147	337	178	141	319	193	143	336
Multiple myeloma (C88+C90)	120	153	273	142	157	299	135	140	275	116	142	258	134	170	304	134	141	275	162	160	322	126	149	275	152	158	310
Non-Hodgkin lymphoma (C82-86, C96)	295	256	551	288	293	581	304	272	576	274	290	564	322	286	608	300	278	578	299	327	626	280	301	581	255	287	542
Oesophagus cancer (C15)	467	129	596	457	118	575	522	105	627	432	112	544	464	113	577	471	87	558	427	104	531	459	89	548	450	103	553
Ovarian cancer (C56)		700	700		700	700		739	739		729	729		727	727		696	696		744	744		716	716		660	660
Pancreas cancer (C25)	942	908	1,850	950	1,053	2,003	943	1,033	1,976	958	1,041	1,999	946	1,032	1,978	1,056	1,122	2,178	1,084	1,144	2,228	1,071	1,082	2,153	982	1,137	2,119
Prostate cancer (C61)	1,198		1,198	1,125		1,125	1,211		1,211	1,280		1,280	1,258		1,258	1,301		1,301	1,389		1,389	1,314		1,314	1,319		1,319
Stomach cancer (C16)	955	746	1,701	1,000	732	1,732	942	677	1,619	904	698	1,602	853	647	1,500	930	649	1,579	845	627	1,472	819	614	1,433	779	554	1,333
Testicular cancer (C62)	53		53	37		37	36		36	58		58	40		40	38		38	43		43	48		48	47		47
Thyroid cancer (C73)	32	71	103	40	65	105	26	61	87	27	60	87	24	55	79	42	43	85	39	74	113	28	46	74	31	56	87
Other	862	968	1,830	807	970	1,777	806	934	1,740	715	889	1,604	734	912	1,646	683	875	1,558	694	839	1,533	744	887	1,631	731	860	1,591

Bold values indicate total case numbers, considering all tumor types included in the study.

### Age-standardized incidence and mortality rates

**Figure 1:**
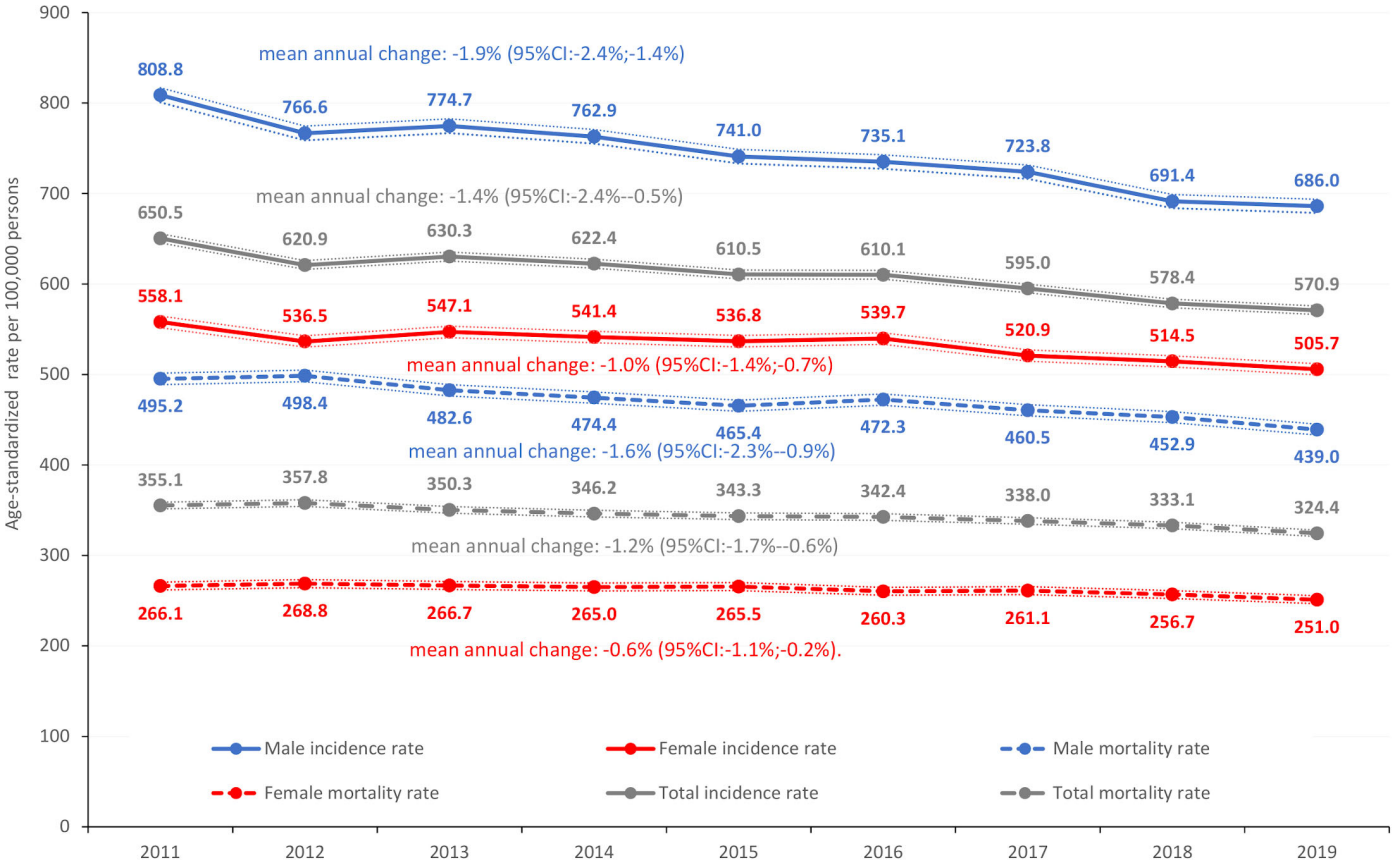
Age-standardized overall cancer incidence rates and overall cause-specific cancer mortality rates in Hungary between 2011 and 2019 (ESP 2013) (per 100,000 PYs; dotted lines represent 95% CI). CI, confidence interval; ESP, European Standard Population; PYs, person-years.

Age-standardized incidence rates of all cancers excluding C44 (ESP 2013) decreased from 808.8 in 2011 to 686.0/100,000 PYs in 2019 in men, with a mean annual decrease of 1.86% (95% CI: 1.36-2.35; decrease for the whole study period: 15.18%) (**Figure 1**). Corresponding numbers in females were 558.1 in 2011 (31.0% lower than in males) and 505.7 in 2019, showing a mean annual decrease of 1.04% (95% CI: 0.66-1.42). Age-standardized overall cause-specific mortality rates (excluding C44) were 495.2/100,000 PYs in 2011 and 439.0/100,000 PYs in 2019 in males, with a mean annual decrease of 1.63% (95% CI: 0.92-2.34) (**Figure 1**). Similarly, mortality rates were 266.1/100,000 PYs in 2011 and 250.98/100,000 PYs in 2019 among females, corresponding to a significant mean annual decrease of 0.64% (95% CI: 0.15-1.13). On the other hand, the annual number of registered cancer deaths did not significantly change during the study period, remaining between 32,000–33,000 in both genders. The overall, combined incidence rate of cancer has increased from 650.5 onto 570.9/100,000 PYs with a yearly average of 1.4% (95%CI: 2.4%-0.5%), while the overall mortality rate has change from 355.1 onto 324.4/100,000 PYs, showing also significant yearly average change (-1.2%; 95%CI:-1.7%–0.6%).

### Age-standardized incidence and mortality rates by most common tumor types in 2018

**Figure 2:**
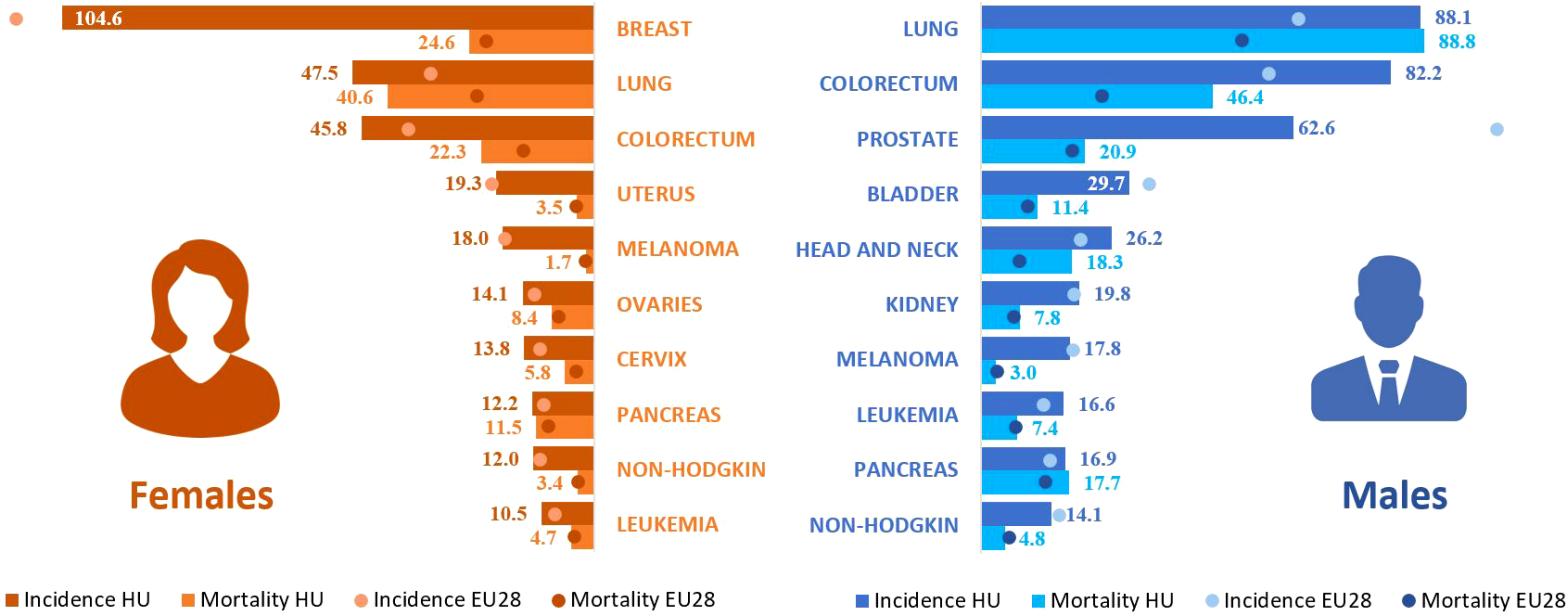
Age-standardized incidence and mortality rates for the 10 most common cancer types in Hungary in 2018 using ESP 1976. Incidence and mortality rates are compared with the European Union (28) average based on Ferlay’s report from 2018 (3). ESP, European Standard Population.

In 2018, the most common cancer type among men was lung cancer, with an age-standardized incidence rate of 88.3/100,000 PYs, followed by colorectal cancer (82.2/100,000 PYs) and prostate cancer (62.3/100,000 PYs). Mortality rates were 88.8, 46.5, and 20.9 per 100,000 PYs, respectively, using ESP 1976 (**Figure 2**). In 2019, the age-standardized incidence of colorectal cancer exceeded that of lung cancer (87.2 vs. 79.3/100,000 PYs, respectively) (**Supplementary Table 4**). Incidence and mortality rates of lung cancer and colorectal cancer were well above the European Union (EU) average.(3) However, the incidence of prostate cancer was lower.

In women, the three most common cancer types were the breast cancer (104.6/100,000 PYs), lung cancer (47.7/100,000 PYs), and colorectal cancer (45.8/100,000 PYs). The highest mortality rate was found for lung cancer (40.6/100,000 PYs), followed by breast cancer (24.6/100,000 PYs), and colorectal cancer (22.6/100,000 PYs) in 2018. Incidences of colorectal cancer and lung cancer were higher, than the EU average; however, breast cancer incidence was similar. **Figure 2** provides a comprehensive overview of the other most common cancer types among males and females, comparing their incidence rates and rankings to the EU average. For international comparisons, age-standardized incidence and mortality rates of different cancer types are shown for the whole study period with ESP 1976 (**Supplementary Table 4**) as well as with ESP 2013 (**Supplementary Table 5**).

### Incidence and mortality of all cancer types in Hungary compared to other European countries

**Figure 3:**
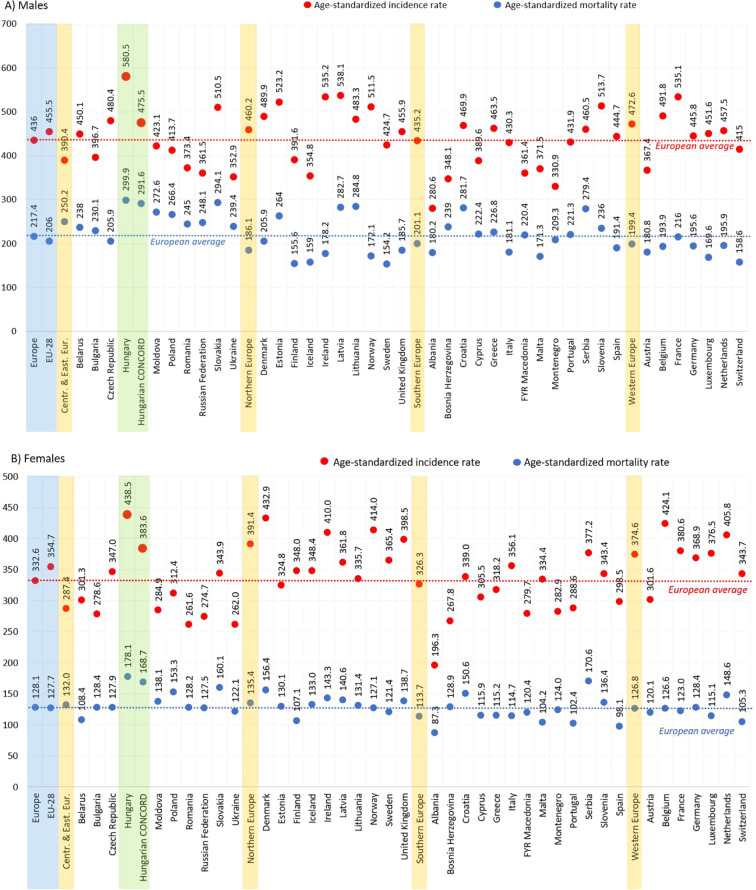
Age-standardized incidence (red) and mortality (blue) rates in males **(A)** and females **(B)** for all cancer types (excluding C44) per 100,000 PYs in European countries and Hungary based on Ferlay’s report (2) and in the HUN-CANCER EPI study using the NHIF/HCSO databases in 2018 (ESP 1976). ESP, European Standard Population; NHIF, National Health Insurance Fund; HCSO, Hungarian Central Statistical Office; PYs, person-years. Description: EU-28: 28 European Union member states in 2018.

In the current study, the age-standardized incidence of all cancer types (excluding C44) was 475.5/100,000 PYs in 2018 in males, which was higher, than the EU-27 average (436.0/100,000 PYs), but significantly lower, than previously estimated for Hungary (580.5/100,000 PYs using ESP 1976) (**Figure 3A**). Incidence rates found in the current study were comparable to those from the Czech Republic, Slovakia, Lithuania, and Estonia, and higher than in Austria, a neighboring but more developed country. The age-standardized mortality rate of all cancer types was 291.7/100,000 PYs in 2018 in males, one of the highest among European countries (average: 217.4/100,000 PYs), but somewhat lower, than in Ferlay’s report (299.9/100,000 PYs) (**Figure 3A**) (3).

In women, the age-standardized incidence of all cancer types (excluding C44) was 383.6/100,000 PYs in 2018, which was higher, than the EU-27 average (332.6/100,000 PYs), but significantly lower, than previously estimated for Hungary (438.5/100,000 PYs using ESP 1976). The age-standardized mortality rate for all cancer types was 168.7/100,000 PYs compared to the European average of 128.1/100,000 PYs in 2018, considerably lower, than the rate estimated by Ferlay et al. (178.1/100,000 PYs) (**Figure 3B**). (3) Mortality rates remained among the highest in Europe in both sexes and were higher than in other Central Eastern European countries.

### Change in incidence and mortality rates of different cancer types between 2011 and 2019

**Figure 4:**
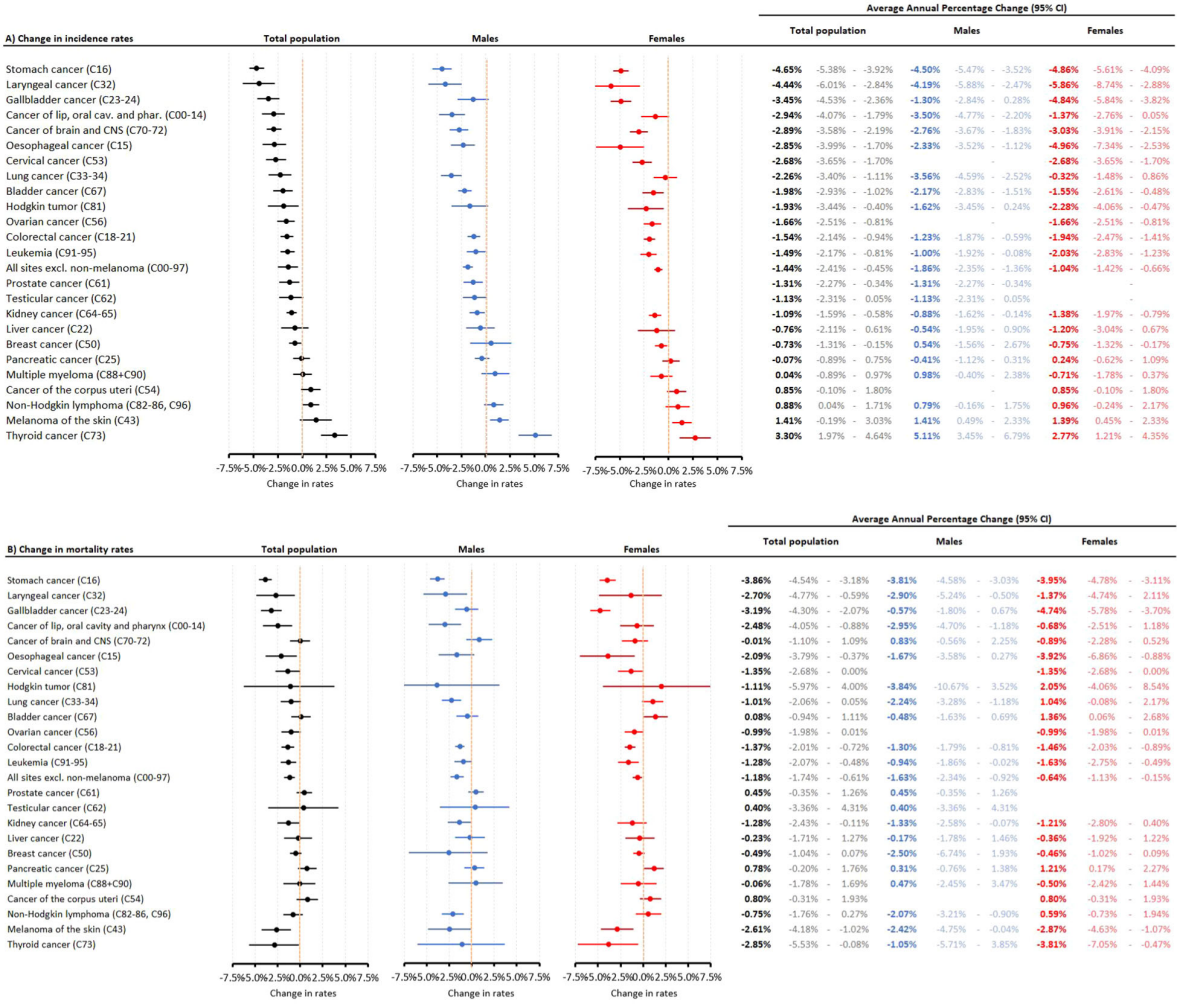
Changes in incidence **(A)** and cause-specific mortality rates **(B)** between 2011 and 2019 (prior to the Covid-19 pandemic) among the most common cancer types in the total study population and according to sex. CI, confidence interval.

**Figure 4** shows the changes in incidence and cause-specific mortality rates of all cancer types (excluding C44) among the most common cancer types between 2011 and 2019.

The highest annual decreases in incidence rates were found in stomach cancer (4.65%), laryngeal cancer (4.44%) and gallbladder cancer (3.45%), while there were significant increases in the incidence of non-Hodgkin lymphoma (0.88%), melanoma of the skin (1.41%) and thyroid cancer (3.30%). The most pronounced decreases in cause-specific mortality rates were found in stomach cancer (3.85%), gallbladder cancer (3.20%), thyroid cancer (2.91%), laryngeal cancer (2.71%), and melanoma of the skin (2.66%). The only significant increases in mortality rates were found among women for bladder cancer (1.38%) and pancreatic cancer (1.22%).

### Change in overall incidence and mortality rates by different age cohorts between 2011 and 2019

**Figure 5:**
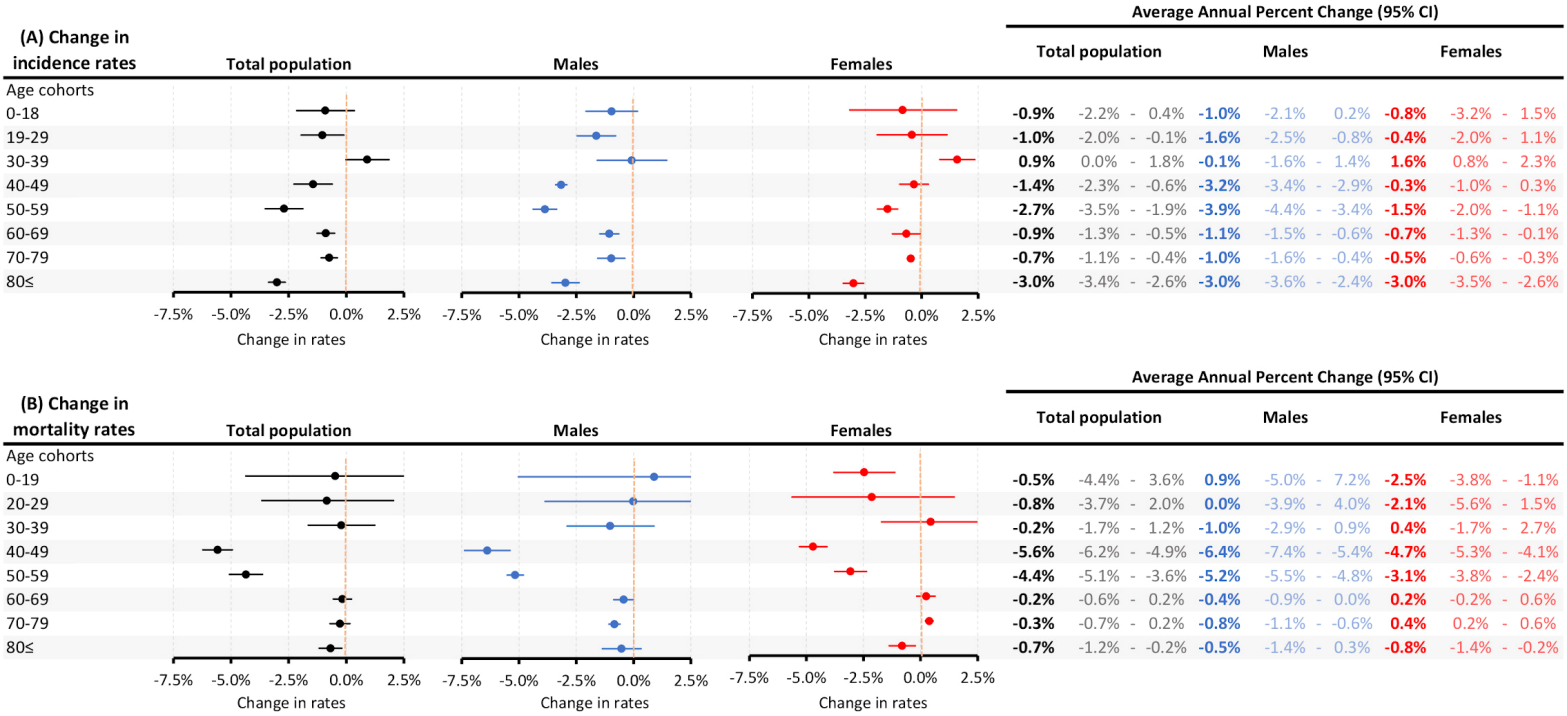
Age and sex related average annual percent changes in incidence **(A)** and cause-specific mortality rates **(B)** between 2011 and 2019 (prior to the Covid-19 pandemic). CI, confidence interval.

**Figure 5** shows the changes in overall incidence and cause-specific mortality rates of all cancer types (excluding C44) among the different age cohorts by different sex, between 2011 and 2019. The most relevant decrease of cancer incidence was found in the 40-49, 50-59 and in 80≤ age cohorts, especially in case of males, where we found a 3.9% average annual decrease (4.4%-3.6%) at the 50-59 cohorts. On the other hand, the increase of average annual incidence was significant in the female 30-39 cohort (1.6%; 95%CI:0.8%-2.4%). The age and sex related change of mortality rates were mostly similar to the incidence rate trends, the highest decrease in the mortality rate was found in the 40-49 and 50-59 male cohorts. The only significant increase in mortality rate was found in the 70-79 cohort of females.

## Discussion

This nationwide, retrospective study was performed as part of the Hungarian HUN-CANCER EPI Multiple Cancer Epidemiology program to assess incidence and mortality rates of all cancer types between 2011 and 2019 in Hungary. Besides the epidemiological data of the Hungarian National Cancer Registry (14, 15), this study provides real-world data on all cancer types in Hungary, based on the analysis of the national health insurance fund database.

The main results of our analysis were as follows:

- 1. Age-standardized incidence rates of all cancer types were significantly lower in both sexes in 2018, than estimates reported by Ferlay et al. (18.1% lower in men and 12.4% lower in women; HUN-CANCER EPI vs. Ferlay et al.: 475.5 vs. 580.5/100,000 PYs in males and 383.6 vs. 438.5/100,000 PYs in females in 2018).

- 2. Age-standardized incidence of all cancer types was higher in males, and significantly decreased by 1.46% on average per year (by 1.86% in males and by 1.04% in females). Age-standardized mortality rates showed a mean annual decrease of 1.16% in males and 0.57% in females between 2011 and 2019. Both decrease of incidence and mortality rates were most pronounced at 40-59 age cohorts of males.

- 3. In 2018, the five most common cancer types were lung, colon, prostate, bladder and head and neck cancers in males, and breast, colon, lung, uterine cancer, and melanoma of the skin in females.

- 4. Age-standardized incidence rates (ESP 1976) were generally higher, than the EU average, except for prostate cancer, where the Hungarian incidence rates were significantly lower.

Recent cancer incidence reports, such as those from the European Cancer Information System (ECIS), Globocan, and Cancer Incidence in Five Continents (CI5), provide comprehensive and up-to-date data on the global burden of cancer. The ECIS focuses on detailed cancer statistics across Europe, highlighting regional variations and trends within the continent. Globocan offers a global perspective, estimating cancer incidence and mortality worldwide, with a particular emphasis on variations between high- and low-income countries. The CI5 series, published by the International Agency for Research on Cancer (IARC), compiles data from cancer registries across five continents, enabling comparisons of cancer patterns and trends over time and across different populations. Together, these sources underscore the importance of continuous monitoring and international collaboration in understanding and addressing the global cancer burden.

However, previous GLOBOCAN estimates on overall cancer incidence in Hungary were solely based on the methodology applied by Ferlay et al. for 2018. Namely, cancer mortality rates reported by the HCSO were used for the calculation of incidence rates. Mortality data from the 2006–2015 period served as a basis for the calculation of age-standardized mortality rates in 2018, already resulting in a slight overestimation. Then, incidence and mortality data from the Czech Republic and Slovakia were derived from their respective cancer registries. As a final step, Hungarian incidence rates were estimated using mortality-per-incidence rate ratios from these two neighboring countries and HCSO-based mortality data. In other words, incidence and mortality rates reported in the Czech Republic and Slovakia were ‘translated’ into Hungarian incidence data (3).

Our recent publication on the epidemiology of lung cancer in Hungary demonstrated the high proportion of only post-mortem diagnosed lung cancer cases in Hungary, accounting for 11.1% of cancer-related cause-specific mortality. This may be due to the much higher autopsy rate compared to other European countries (7, 8). Lower autopsy rates for hospital deaths decrease the likelihood of post-mortem cancer diagnoses, especially for cancers commonly diagnosed in late stages. Therefore, cause-specific mortality reported from countries with low autopsy rates may be lower than the actual numbers. The Czech Republic and Slovakia were reported to have 16.6% and 10.9% autopsy rates of hospital deaths in 2018, respectively, compared to the Hungarian rate of 35.8% (8). Traditionally, international comparisons of mortality rates have used HCSO-derived data from Hungary, which contains post-mortem diagnosed cases. Therefore, we propose using NHIF-based cancer incidence data from Hungary for comparisons of cancer burden with other European countries, at the same time acknowledging that actual cancer incidence may be higher due to the significant proportion of post-mortem diagnosed cases. Of note, autopsy rates significantly decreased during the study period in Hungary, which may partly explain the decrease of standardized mortality. On the other hand, the change in age distribution in Hungary during the study period may also have contributed to the observed decrease in standardized mortality rates, which is supported by the fact that the number of deaths did not change during the study period. Incidence rates found in our study are much more in line with those reported from other post-socialist countries in the region, than shown by previous estimates. Cancer incidence in Hungary was found to be in a similar range to that reported from the Czech Republic, Slovakia, and Serbia based on their respective cancer registries, and much higher, than incidence rates reported from Austria, a neighboring but more developed country. This similarity can be attributed to the comparable prevalence of cancer risk factors in these countries. Smoking is one of the main risk factors for the development of cancer (16). The estimated proportion of regular daily smokers in the population aged 15 years and over was 25.8% in 2014 in Hungary, which only slightly exceeds the EU average (22.3%) and proportions in the Czech Republic (22.3%), Slovakia (22.9%), and Austria (24.3%), and is lower than in Croatia (27.5%), Bulgaria (28.2%), and Serbia (29.2% in 2013) (WHO) (17). In terms of obesity, which is another important risk factor for cancer development (18), Hungary ranks among the countries with the highest prevalence of overweight (58.4% in 2014 vs. 51.2% EU average), with similar rates observed in the Czech Republic (58.4%), Slovakia (57.8%), Poland (56.7%), and Croatia (63.8%). Regular alcohol consumption is also known to increase the risk of cancer (19). In Hungary, the proportions of daily and weekly alcohol consumers were 6.3% and 19.1% in 2019, respectively, lower than the EU average (8% and 28.4%, respectively), Czech Republic (7.8% and 33.8%), and Bulgaria (10.2% and 23.8%), but similar to Slovakia (4.1% and 19.3%), Croatia (10.2% and 17.9%), and Romania (2.9% and 19.0%) (20).

After conducting a thorough validation of the HUN-CANCER EPI study data on incidence and mortality, we can state that the incidence of diagnosed cancer in Hungary is higher, than the European average in both sexes, albeit not exceptionally high compared to other Central European countries. Of note, based on analysis, these rates showed a decreasing trend over the past decade, even prior to the onset of the Covid-19 pandemic (21, 22). Our results highlight a fundamental shift in perception about cancer burden in Hungary, showing similar cancer incidence rates to other countries in the region, rather than exceptionally high incidence rates in Europe. These findings support the need for the unification of data collection and adjustment guidelines which may establish more accurate and comprehensive cancer registries in post-socialist countries. Achieving this goal would allow for the more accurate analysis of cancer burden in the region, since the role of population-based registries is essential to provide reliable epidemiological data. Until then, currently available cancer incidence estimates, including our current study, should be interpreted with caution, acknowledging their limitations (3).

According to the population-based data of GLOBOCAN, in the majority of EU member states the standardized incidence rates of all malignant tumors excluding C44 stagnated or decreased in 2011-2018 or in part of the period for which data were reported (23). This is in line with our findings. Nevertheless, the studied period was relatively short, and the future availability of longer time series will allow for the observation of more reliable trends over time. Beside the already implemented prevention and screening programs, to ensure the positive trends in cancer incidence, more focus should be put on the Third Expert Report on Diet, Nutrition, Physical Activity, and Cancer by the World Cancer Research Fund (WCRF), the European code against cancer guidelines and the American Institute for Cancer Research (AICR), which offers a comprehensive analysis of current research in cancer prevention and survivorship (24). It provides a global framework for reducing cancer burden, enhancing health, and improving the quality of life for survivors, while guiding healthcare practitioners on diet and lifestyle changes. Additionally, it outlines research priorities to improve evidence-based recommendations for public health and personalized strategies for at-risk individuals and cancer survivors.

Our study has important strengths and limitations that need to be considered when interpreting the results. One of the notable strengths lies in the robust number of identified cancer patients, which increases the statistical reliability of our findings. The data were thoroughly cleaned, ensuring their accuracy and validity. Additionally, the extended 9-year follow-up period provided a comprehensive view of cancer trends over time. The nationwide coverage of the NHIF database and its comparison to HCSO mortality data allowed for a more comprehensive assessment of cancer outcomes in the country. Furthermore, the inclusion of cancer-related interventions helped to exclude cases where cancer-associated ICD codes were used mistakenly, and the interventions did not align with the ICD code and the patient did not die shortly after the ICD code assignment. These methodological considerations contribute to the soundness of our conclusions.

However, there are certain limitations, as well. Our reported incidence rates are generally lower than those published by the Hungarian National Cancer Registry, which is due to the different data cleaning and processing methods. For example, one reason for the difference is that the registry contains each reported case until the rapporteur withdraws it in case of false reporting (i.e., the diagnosis of the case changes), whereas our study used the rules for case definition described in the Methods section. Furthermore, the NHIF database lacks information on post-mortem diagnosed cases, which could have resulted in the underestimation of cancer incidence. The impact of post-mortem diagnosed cancer cases highly depends on the autopsy rate for hospital deaths. While this limitation exists, it is worth noting that the autopsy rate in most other European countries is generally below 10%. Therefore, the potential underestimation of cancer incidence due to the exclusion of post-mortem identified cases might be of a similar magnitude, rendering our results still comparable to those of other countries. Another limitation is related to the methodology of identifying cancer patients based on the majority of cancer-related ICD code records. Although a sensitivity analysis was performed, this approach might lead to the exclusion of patients who could have had secondary or multiple primary tumors, potentially resulting in a certain underestimation of cancer incidence. Moreover, our retrospective database analyses span a 9-year period, during which patients initially diagnosed with a certain primary tumor could have developed another type of primary cancer during the follow-up period. This temporal aspect should be considered when interpreting the results and understanding the potential changes in cancer diagnoses over time. Previous analyses from the SEER Program in 1995 reported that multiple primary neoplasms accounted for 13.1% of cancers in men and 13.7% in women (25, 26). In a study from Portugal, the 5-year cumulative incidence of secondary primary cancer was 3.0% (27). In our 9-year study, we estimated approximately 14,000 cases of secondary primary cancer over a median follow-up of 4.5 years in a prevalent cancer population of 459,204 individuals in 2019 (including incident cases in 2019). This may have led to an underestimation of around 1,500 new cancer cases annually (2.7%). Moreover, the NHIF database lacked information on molecular histology, TNM stage, and ECOG status of the patients, preventing us from assessing cancer incidence by specific histological and molecular subtypes and examining the impact of patient-related factors on cancer mortality. We are also emphasizing, that there is a cancer registry for childhood malignancies available in Hungary led by Semmelweis University, which reports cancer incidence and survival estimations for 0-18 year cohort on a regular basis.

Given that cancer incidence trends can be sensitive to the duration of the screening period (2 years from 2009 and 2010), we conducted a trend analysis with the use of a longer, six-year screening period. We calculated the average yearly incidence change from 2015 to 2019 and observed a significant decrease, greater than that seen in the 2011-2019 period, for both sexes. For males, the average yearly decrease was 2.10% (95% CI: 1.47%-2.72%-), and for females, it was 1.67% (95% CI: 1.18%-2.17%-). Regarding mortality rates, the 2015-2019 period also showed a significant decrease, slightly higher than the previous period, with a yearly average of 1.67% (95% CI: 0.48%-2.85%-) for males and 1.14% (95% CI: 0.33%-1.94%-) for females. Therefore, the longer screening period confirmed that cancer incidence and mortality rates have significantly decreased for both sexes in Hungary over the last decade.

## Conclusion

The HUN-CANCER EPI study represents an effort to provide more accurate estimates of the incidence of newly diagnosed cancer cases in Hungary using the database of the Hungarian National Health Insurance Fund in a pioneering way. Our results show a significantly lower number of diagnosed cancer cases compared to previous GLOBOCAN estimates which were based on cause-specific cancer mortality data and mortality-to-incidence rate ratios from neighboring countries. One crucial factor contributing to the previous overestimation of cancer incidence in Hungary is the exceptionally high autopsy rate for hospital deaths in the country which led to a high number of post-mortem diagnosed cancer cases coded as the underlying cause of death, thus inflating incidence estimates. Our newly calculated cancer incidence rates are consistent with data from other Central Eastern European countries in line with the similar prevalence of major cancer risk factors in these countries. The highest incidence rates were found for lung, colon, and prostate cancer in men, and for breast, colon, and lung cancer in women, which is consistent with international findings. Furthermore, our study revealed decreasing trends in cancer incidence and mortality for almost all cancer types. These trends can largely be attributed to the decreasing prevalence of smoking, driven by European and Hungarian initiatives and regulations aimed at tobacco control. Nevertheless, the burden of cancer is still very high in Hungary, and its control should be a public health priority. The means are known and should be implemented.

## Data Availability

All data presented in the manuscript can be found directly in the **Supplementary Material**.
